# Assessing the Heat Tolerance of Meiosis in Spanish Landraces of Tetraploid Wheat *Triticum turgidum*

**DOI:** 10.3390/plants11131661

**Published:** 2022-06-23

**Authors:** Tomás Naranjo, Nieves Cuñado, Juan Luis Santos

**Affiliations:** Departamento de Genética, Fisiología y Microbiología, Facultad de Biología, Universidad Complutense de Madrid, 28040 Madrid, Spain; nicuna@bio.ucm.es (N.C.); jlsc53@bio.ucm.es (J.L.S.)

**Keywords:** heat stress, chiasmata, durum wheat, genetic diversity

## Abstract

Heat stress alters the number and distribution of meiotic crossovers in wild and cultivated plant species. Hence, global warming may have a negative impact on meiosis, fertility, and crop productions. Assessment of germplasm collections to identify heat-tolerant genotypes is a priority for future crop improvement. Durum wheat, *Triticum turgidum*, is an important cultivated cereal worldwide and given the genetic diversity of the durum wheat Spanish landraces core collection, we decided to analyse the heat stress effect on chiasma formation in a sample of 16 landraces of *T. turgidum* ssp. *turgidum* and T. *turgidum* ssp. *durum*, from localities with variable climate conditions. Plants of each landrace were grown at 18–22 °C and at 30 °C during the premeiotic temperature-sensitive stage. The number of chiasmata was not affected by heat stress in three genotypes, but decreased by 0.3–2 chiasmata in ten genotypes and more than two chiasmata in the remaining three ones. Both thermotolerant and temperature-sensitive genotypes were found in the two subspecies, and in some of the agroecological zones studied, which supports that genotypes conferring a heat tolerant meiotic phenotype are not dependent on subspecies or geographical origin. Implications of heat adaptive genotypes in future research and breeding are discussed.

## 1. Introduction

Cultivated wheats represent the staple food of more than 35% of the world’s population and provide one fifth of the daily intake of calories and protein [1]. Most of the global wheat production, 760.93 million metric tons in 2020 (http://www.fao.org/faostat/es/#data/QC accessed on 8 April 2022), corresponds to bread wheat (*Triticum aestivum* L.), the production of durum (pasta) wheat (*T. turgidum* L.) has been estimated in 5% of the total wheat amount [2]. Wheat yield improvement becomes a priority of the breeding programs in order to meet the global demand expected for the next decades propelled by world population increase.

Durum wheat is the cultivated form of *T. turgidum,* an allotetraploid species (2n = 4x = 28) with genome formula AABB, which is believed to have originated between the past five hundred thousand years [3] after hybridization between *T. urartu*, the A genome donor, and a species close to the extant *Aegilops speltoides* that contributed the B genome [4,5,6]. Bread wheat, *T. aestivum* is an allohexaploid species (2n = 6x = 42, genome formula AABBDD) originated after a second hybridization event, most likely occurred under cultivation about 10,000 years ago, between durum wheat and *Ae. tauschii* the wild diploid progenitor of the D genome [7,8]. Despite the coexistence of two genetically related genomes in durum wheat, and three in bread wheat, both polyploid species show a strict diploid-like meiotic behaviour at metaphase I, with 14 and 21 bivalents, respectively, formed after recombination between homologous chromosomes. Formation of chiasmata in polyploid wheats is driven by the action of promoter and suppressor genes [9]. Recombination between homoeologous chromosomes is impeded by the action of the most effective pairing suppressor, the *Ph1* locus located on the long arm of chromosome 5B [10,11,12,13,14,15,16,17].

Meiotic recombination is an elaborate process that, in plants, takes place over a time lapse of many hours during the first meiotic division and involves numerous steps [18,19]. The meiotic recombinational machinery develops an important DNA repairing process that is triggered by the occurrence of programmed double strand DNA breaks at the commencement of meiosis [20]. Initial recombination steps can progress through different pathways in such a way that only a minor fraction of all recombinational events matures as crossovers that culminate in chiasmata [20].

Meiosis, as well as many other biological processes in plants, is extremely sensitive to adverse climatic environments. Abiotic stresses, such as low and high temperatures, salt stress, osmotic shock and water deficit, have a negative impact on male gamete development and cause a considerable reduction of male fertility [21,22]. The rise of temperature derived from the climate change is, therefore, an abiotic stress factor with potential effects on meiosis. Suboptimal high and low temperatures and their effects on crossover frequency and distribution have been investigated in both wild and cultivated plant species [23]. In most instances, variations in the crossover landscape were inferred from chiasmata observed at metaphase I. A consistent reduction of the mean chiasma frequency with increasing temperature was reported in *Endymion nonscriptus* [23,24] and *Rhoeo spathacea* [25]. High temperature from 34 °C to 41 °C reduces also chiasma frequency in *Tradescantia bracteata* and *Uvularia perfoliata* [26]. In contrast, the rise of temperature increases chiasma frequency in rice [27] and barley [28]. In *Arabidopsis thaliana*, recombination frequencies variation in response to temperature follows a U-shaped curve with the highest values at 8 °C and 28 °C, and the lowest one at 18 °C [29]. Modification of chiasma distribution has also been documented in some of the above-mentioned species [27,29].

In *T. aestivum*, the optimum range of temperature throughout its entire growing season is considered to be around 17–23 °C [30]. Bread wheat is less sensitive to temperature increase during its vegetative phase than during its reproductive phase [31]. High temperatures during reproductive development cause a detrimental effect on grain yield [31,32], a treatment of 24 h at 30 °C during meiosis is sufficient to reduce grain number [33,34]. In the bread wheat variety Chinese Spring, meiosis was estimated to take 24 h at 20 °C, but this duration is reduced by several hours at higher temperatures [35,36,37]. Temperature increase causes a reduction in chiasma frequency in bread wheat [38] while other meiotic irregularities such as laggards, chromosome bridges, micronuclei disturbances, chromatin pulling or abnormal cytokinesis are produced in *T. turgidum* [39]. Bayliss and Riley [38] studied the position and duration of the temperature-sensitive stage of hexaploid wheat in plants nullisomic 5D-tetrasomic 5B (N5DT5B). Plants lacking chromosome 5D show a reduction in chiasma frequency at temperatures below 15 °C or above 29 °C and normal chiasma frequencies at intermediate temperatures. The temperature-sensitive stage occurred in the premeiotic interphase, prior to DNA synthesis [40]. The chiasma number reduction is a result of synapsis failure occurred in zygotene [38,41]. The meiotic recombination gene *Dmc1* located on chromosome 5D has been identified as a candidate for the maintenance of normal chiasma frequency at low and possibly high temperatures in hexaploid wheat [42]. As a AABB tetraploid, the D copy of *Dmc1* (*Dmc1-5D*) does not exist in durum wheat contrary to the A and B copies (*Dmc1-5A* and *Dmc1-5B*, respectively) that are present in both the tetraploid and hexaploid species [42].

Nowadays landraces are considered as a natural reservoir of the genetic variation within the species and one of the most important sources for potentially favourable alleles to be used in breeding programs. Core collections of germplasm, where genetic diversity is maximized with minimum repetition, are a favoured approach to efficiently exploring novel variation and enhancing the use of germplasm collections. The core collection of Spanish landraces of durum wheat is characterised by a high genetic diversity with genetic diversity Dice indices of 0.79 for a sample of 39 SSR loci and 0.84 for gliadin loci [43]. Such values are higher than those reported for other durum wheat collections [44] denoting a considerable level of polymorphism among the Spanish durum wheat genotypes. Three subspecies, *T. dicoccon*, *T. turgidum*, and *T. durum*, comprising 13, 38, and 139 genotypes, respectively are present in the core collection. Genotype variation is lower in *T. dicoccon* and *T. turgidum* than in *T. durum* while genetic differentiation by the agroecological zone of origin is greater in *T. dicoccon* and *T. turgidum* than in *durum* [44]. 

The aim of this paper was to explore the genetic variability of landraces from the Spanish durum wheat core collection in order to verify the existence of thermotolerant genotypes capable of developing regular meiosis at high temperature. We have analysed a sample of 16 landraces belonging to the two subspecies, *T. triticum* and *T. durum,* with the highest number of different genotypes, from localities spread through all of the Spanish territory with variable climate conditions. Chiasma formation has been studied at permissive and restrictive (high) temperatures in the 16 landraces of durum wheat, to assess the response of each genotype to heat stress.

## 2. Results

### 2.1. Characteristics of the Spanish Landraces Studied

Landraces of durum wheat analysed were sampled from all of the nine agroecological zones in which the Spanish territory was divided. These zones were defined by Ruiz and coworkers [44] on the basis of historical yield records and climatic condition. Geographic location and altitude of the collecting site of all 16 genotypes analysed as well as their names and subspecies, *T. durum* (d) or *T. turgidum* (t), assignation appear in Table 1.

**Figure 1:**
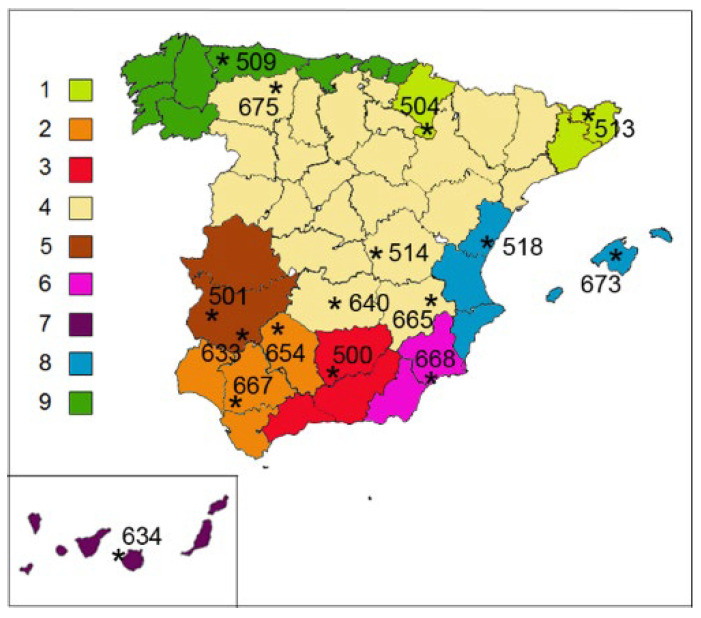
The collecting site location (⁎) of 16 Spanish landraces of *T. turgidum* in nine (1–9) agroecological zones of Spanish territory represented with different colours. The three numbers located next to each locality are the last three digits of the corresponding accession (first column of Table 1).

The location of the nine agroecological zones in the map of Spain as well as the situation of the collecting localities of the 16 landraces studied are shown in Figure 1. The situation of each locality (Village, Table 1) in the map of Spain is indicated by an asterisk, the three numbers located next to each locality are the last three digits of the corresponding accession (first column of Table 1). Zone 4 occupying the centre and part of the northeast of the Iberian Peninsula covers by far the largest territory of the nine zones established, reason why four genotypes were studied in zone 4 and one or two in the remaining conditions.

### 2.2. Chiasma Analysis

**Figure 2:**
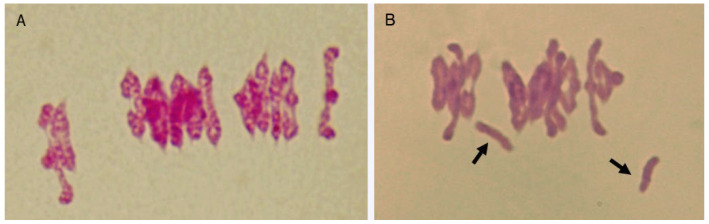
Cells of *T. turgidum* at metaphase I. (**A**) 12 ring bivalents and two rod bivalents (26 bound arms). (**B**) Nine ring bivalents, four rod bivalents and two univalents (arrowed) (22 bound arms).

Unlike what happens in other organisms, chiasma identification at diplotene and diakinesis in durum wheat has associated problems due the difficulty in distinguishing relational twists from genuine chiasmata. Therefore, metaphase I is a more suitable stage for chiasma scoring in this species despite the condensed state of the chromosomes. Durum wheat chromosomes are metacentric or submetacentric, thus a number of 28 bound chromosome arms (14 ring bivalents) is expected when chiasmata occur in all chromosome arms. Whereas, rod bivalents are bound by chiasmata in only one arm, univalents are the result of the absence of bonds between homologues. In the analysis performed here we assume the existence of a single chiasma per bound arm since no cytological evidence of the presence of a second chiasma was found. Nevertheless, the possibility of more than one chiasma per chromosome arm in some bivalents cannot be ruled out. Examples of two PMCs at metaphase I after Feulgen staining are shown in Figure 2.

For each genotype and treatment, the number of ring bivalents, rod bivalents and univalent pairs, as well as the number of bound arms per cell were scored. The mean number of bound arms per cell and the frequency of cells with two univalents are indicated in Table 2. Plants used as control were grown in the greenhouse at the optimum temperature range (18–22 °C) and showed normal meiosis. Between lines differences in the number of bound arms per cell were significant (*p* < 0.001) when a Kruskal-Wallis test was used. The number of bound arms per cell was higher than 27 in 12 genotypes. On average these genotypes formed 13 ring bivalents plus one rod bivalent, two of then showed also one cell with two univalents. Four accessions, BGE047504, BGE045668, BGE045634, and BGE045673, formed chiasmata that were in the range between 26 and 27 bound arms per cell, all of them with some cell showing two univalents.

Plants subjected to heat treatment were moved from the greenhouse to a climatic chamber at 30 °C when their spikes were at premeiosis (see Material and Methods). Heat treatment was applied for a minimum of 48 h, time sufficient to ensure that the premeiotic temperature-sensitive stage was affected. The spikes of the same plant were not synchronous and differed in the degree of development when heat treatment was applied. Once a given plant started the heat treatment, spikes assumed to be on meiosis after a minimum of 48 h were collected to identify the meiotic stage of their anthers under light microscopy. Cells at metaphase I were found in only 20% of spikes, and other meiotic stages, mainly prophase I and tetrads, were observed in the remaining 80%. In order to get cells at metaphase I from all landraces, treated plants were maintained at 30 °C until a sufficient number of anthers with meiocytes at metaphase I were recovered. Spikes collected after 48 h of treatment contained meiocytes at metaphase I in four landraces. In the remaining landraces, metaphase I was found in younger spikes collected after heat treatments of more than 48 h, which appear indicated in Table 2.

The average number of bound arms per cell in the control plants grown at optimum temperature range (18–22 °C) and heat-treated plants, as well as the variation produced by the heat treatment, are shown in Table 2. Heat treatment caused no reduction of chiasma frequency in accessions BGE045654 and BGE045668 (zones 2 and 6, respectively, ssp. *durum*), a reduction lower than 0.5 chiasmata in accessions BGE047513 (zone 1, ssp. *triticum*) and BGE045634 (zone 7, ssp. *durum*), a reduction comprised between 0.5 and 1 chiasmata in accessions BGE047500 (zone 3, ssp *durum*), BGE045665 (zone 4, ssp. *durum*), BGE047501 (zone 5, ssp. *triticum*) and BGE047509 (zone 9, ssp. *triticum*), a reduction between one and two chiasmata in accessions BGE045640 (zone 4, ssp. *triticum*), BGE047514 (zone 4, ssp. *durum*), BGE045675 (zone 4, ssp. *durum*), BGE045633 (zone 5, ssp. *durum*), and BGE045673 (zone 8, ssp. *durum*), and a reduction higher than two chiasmata in accessions BGE047504 (zone 1, ssp. *triticum*), BGE045667 (zone 2, ssp. *durum*), and BGE047518 (zone 8, spp. *triticum*). Reduction in the number of chiasmata was mainly a result of the increase of rod bivalents as well as of univalent pairs as a result of failure of chiasma formation in one or both arms of the same homologous pair, respectively. Nevertheless, more than two univalents per cell were rare.

In order to verify whether the differences between heat-treated plants and control plants were significant or not, the number of bound arms per cell in plants grown at 18–22 °C and plants grown at 30 °C were compared using Mann-Whitney-Wilcoxon tests. These comparisons are shown in Table 3. Significant differences between chiasmata numbers are apparent in 13 landraces with a reduction higher than 0.3 in the mean chiasma frequency at 30 °C. However, there is no effect of the temperature increase in the number of bound arms per cell in accessions BGE047513 (ssp. *turgidum*), BGE045654 (ssp. *durum*) and BGE045668 (ssp. *durum*) of zones, 1, 2, and 6, respectively. Thus, meiosis of 19% of the landraces studied tolerates the temperature increase. Among the remaining 13 temperature-sensitive genotypes, BGE047504 (zone 1, ssp. *turgidum*), BGE045667 (zone 2, ssp. *durum*) and BGE047518 (zone 8, ssp. *durum*) showed the highest reduction, more than two chiasmata per cell.

## 3. Discussion

The core collection of Spanish landraces of durum wheat, to which the 16 genotypes analysed belong, is characterised by a high genetic diversity, subspecies *T. durum* showing the highest degree of genetic variability compared to subspecies *T. turgidum* and *T. dicoccon* [44]. The sample of landraces analysed in this work was formed by six genotypes from *T. turgidum* ssp. *turgidum* and ten from *T. turgidum* ssp. *durum*. High temperature-resistant genotypes belong to both subspecies, one (BGE047513) to *T. turgidum* and two (BGE045654 and BGE045668) to *T. durum,* as well as to different agroecological zones, 1, 2, and 6, respectively. Likewise, the most temperature-sensitive genotypes appear in both subspecies, BGE047504 in *T. turgidum* and BGE045667 and BGE047518 in *T. durum,* zones 1, 2, and 8, respectively. Zones 1 and 2 are located in opposite regions of Spain, North-East and South-West, respectively, and show very different climatic conditions, a higher annual rainfall and much lower temperature in zone 1 than in zone 2 [44]. On the other hand, resistant genotypes appear at different altitude and the same happens with temperature sensitive genotypes (Table 1). These findings support that thermotolerance of meiosis in the different landraces is not restricted to a given subspecies or geographical origin.

Differences between heat-treated plants in the number of hours growing at 30 °C, were the result of the short duration of metaphase I [35] compared to longer stages, such as prophase I or tetrads, which were preferentially found in spikes with anthers in meiosis. Among the three heat tolerant genotypes, anthers with cells at metaphase I were collected at 48 h in accessions BGE045654 and BGE045668, and at 96 h in BGE047513. Likewise, the number of hours at 30 °C varied also among the highly temperature-sensitive genotypes: 64, 96, and 120 h, for accessions BGE047504, BGE045667, and BGE047518, respectively, as well as among the ten remaining genotypes. The longest heat treatment, 144 h, was applied in accession BGE045665. Anthers of spikes subjected to this treatment showed meiocytes at different development stages denoting progression of meiosis as in shorter treatments. Thus, regardless of the heat treatment duration, tolerance or sensitivity to the treatment seem to be not dependent on the number of hours that plants were growing at 30 °C. All landraces were grown at 30 °C during the temperature-sensitive stage of the premeiotic interphase [40] and variation in time of the treatment affected development stages prior to premeiotic interphase. Up to date, there is no report supporting that some development stage of germline cells prior to premeiotic interphase is temperature-sensitive and affects chiasma formation.

The absence of disturbed meiosis is essential to achieve a high yield in crop cereals. Meiotic irregularities produced by structural chromosome rearrangements, polyploidy and specific gene mutations cause a reduction of fertility [17,45]. The reduction of up to 50% in grain number produced in the CRISPR *Tazip4-B2* bread wheat is a representative example of the negative impact of meiotic disturbances on a particular agronomical trait [17]. Even though there is no data in durum wheat linking meiosis under the heat stress condition with important agronomic traits, there is evidences of this relationship in bread wheat. Thus, a heat treatment of 30° during meiosis caused a drastic reduction in grain set, but no effect at other development stages. [33,34]. A light microscopy study revealed an abortive development of anthers leading to male sterility (although the meiotic process was not analysed) [46].

Plant heat response is a very complex trait; various plant organs, in a definite hierarchy and in interaction with each other, are involved in determining crop yield under stress [47]. In several crops, including wheat, heat shock proteins synthetized as a response to high temperature stress may protect tissues resident proteins from denaturation [48]. Plants respond also to heat stress by synthetizing enzymes, such as superoxide dismutases, peroxidase and catalase, that contribute to maintain membrane stability by removing reactive oxygen intermediates that accompany temperature increase [49]. Multiple genes are most likely involved in the plant heat stress response but little is known about the role of individual genes in wheat controlling thermotolerance [50]. In addition to the *TaDmc1-D1* gene [42], the TaHsfA2d seed preferential heat shock transcription factor has been suggested to be involved in wheat thermotolerance, since overexpression of TaHsfA2d confers tolerance towards high temperature, as well as to salinity and drought stresses, in transgenic *Arabidopsis* plants [51]. Another gene, *TaB1-1.1*, encoding Bax inhibitor-1 (BI-1) a cell death suppressor conserved in plant and animals, interacting with protein TaFKBP62 mediates also in the response to heat stress [52]. In a recent study, 1413 double haploid lines of bread wheat were screened to assess the genetic basis of heat-stress adaptation. Plants were subjected to three consecutive days of eight hours under an air temperature of 36 °C and a wind speed of 40 km h^−1^. QTL mapping identified nine QTLs correlated to heat stress responsiveness [53]

The global warm coupled with the genetic erosion of wheat germplasm impose the need of finding novel and useful genotypes to be included in crops breeding. Our result that 19% of the durum wheat landraces sample analysed carry genotypes, providing a regular meiotic development at high temperature, confirms the existence of genetic variability among varieties of the Spanish core collection and has potentially high significance. Tolerance of meiosis to heat stress is a good prerequisite to obtain higher yields despite the temperature increase derived from the climate change. The finding of heat tolerant genotypes in the analysed sample represents an incentive to extend the analysis of meiotic thermotolerance to all other varieties of the core collection in purpose of the ulterior identification of genes underlying this response. On the other hand, thermotolerant genotypes the more numerous they are, the better expectations they offer to be used in future breeding programs of durum wheat.

## 4. Materials and Methods

### 4.1. Plant Material

Seeds of 16 landraces of the Spanish core collection of tetraploid wheat *T. turgidum*, six of subspecies *T. turgidum* and ten of subspecies *T. durum*, indicated in Table 1 were obtained from Centro de Recursos Fitogenéticos, Instituto Nacional de Investigación y Tecnología Agraria y Alimentaria (INIA), Spain. These seeds were germinated on wet filter paper in Petri dishes, sowed in pots, and grown in a greenhouse at temperatures between 18 °C and 22 °C. Six plants per cultivar, were maintained in the greenhouse during meiosis, and constituted the control sample. A second sample of six plants per cultivar were transferred from the greenhouse to a cabinet at 30 °C for the premeiotic heat treatment.

### 4.2. Heat Treatment

Plants of the different genotypes, when showed visible the flag leaf ligule, were transferred to a growing cabinet of 4.80 × 2.60 × 2 = 24.96 m^3^ at 30 °C, with air flux to homogenise the temperature, 70% humidity, and a 16-h photoperiod (lights on between 8.00 and 24.00). Plants containing three, four or five erected tillers with an average height of 70 cm, remained in these conditions for a time lapse between 48 h and 144 h, until anthers with meiocytes at metaphase I were collected. A maximum of 24 plants were growing at the same time in the cabinet. Plant pots were kept in trays of water to prevent dehydration. Wheat meiosis takes around 24 h to complete at 20 °C but high temperature speeds up the process [35,36]. A minimum treatment of 48 h at 30 °C of the anthers containing PMCs at metaphase I, ensured that all of them were exposed to heat stress during the temperature-sensitive premeiotic stage.

### 4.3. Sampling of Meiotic Cells and Cytological Analysis of Chiasmata at Metaphase I

Meiosis in wheat occurs when the immature spike is still inside the tiller. External features of the tiller, such as emerged flag leaf and the spike enclosed within the sheaths of the leaves just in the internode between the leaves preceding the flag leaf, help to recognize when the spike was in meiosis. The spike length inside the tiller can be estimated by feeling gently with the fingers its bottom and top end. The immature spike was considered in meiosis when it reached a length close to 5 cm. Every morning spikes assumed to be at meiosis were cut and used for the meiotic stage screening. The first four tillers of each plants were only used. Young spikes collected were carefully dissected to isolate anthers from the two largest florets in each spikelet. The three anthers within any floret are synchronised in meiotic development, so, to determine the meiotic stage one anther from each floret was stained with acetocarmine and squashed under a cover slip to extrude the PMCs, which were then examined using an Eclipse E400 Nikon microscope. When PMCs at metaphase I were identified, the two remaining anthers of the same floret were fixed in 3:1 (*v*/*v*) 100% ethanol/acetic acid and stored at 4 °C for a minimum of two weeks. Fixed anthers were hydrolysed with 1 M hydrochloric acid for 12 min at 60 °C, Feulgen-stained with Schiff’s reagent for a minimum of 30 min, and squashed in 45% acetic acid. Preparations containing PMCs at metaphase I were examined using the Eclipse E400 Nikon microscope, equipped with a Nikon Digital Camera DXM1200F, to score the number of bound arms per cell. With some exceptions, 100 PMCs per landrace and treatment were analysed. For each PMC, the number of ring bivalents, open bivalents and univalent pairs, as well as the number of chromosome arms being bound, were scored. Statistical analysis was performed using the R Software [54]. First, the normality of the distribution of bound arm per cell in heat treated and untreated plants was checked by a Shapiro-Wilk test. Given the absence of normality (*p* < 0.05) in all samples, chiasma formation in the 16 landraces growing in standard conditions was analysed by the Kruskal-Wallis test. The effect of heat treatment was analysed by comparing the number of bound arms per cell of the control and treated plants using Mann-Whitney-Wilcoxon tests.

## Figures and Tables

**Table 1:** Name, collecting site, altitude, subspecies, *T.* *durum* (d) or *T.* *turgidum* (t), and agroecological zone of the 16 Spanish landraces of *T. turgidum* studied.

Accession	Name	Village	Province	Altitude (m)	Ssp.	Zone
BGE047504	Blanco de Corella	Corella	Navarra	373	t	1
BGE047513	Radondell	Les Llosas	Gerona	970	t	1
BGE045667	Rojo de Lebrija	Lebrija	Sevilla	37	d	2
BGE045654	Rubio de Espiel	Espiel	Córdoba	561	d	2
BGE047500	Alcalá la Real	Alcalá la Real	Jaén	1033	d	3
BGE045665	Claro de Higueruela	Higueruela	Albacete	1020	d	4
BGE045640	Torralba de Calatrava	Torralba de Calatrava	Ciudad Real	620	t	4
BGE047514	Torrelengua	Pozorrubio	Cuenca	786	d	4
BGE045675	Blanquillon de Boñar	Boñar	León	979	d	4
BGE047501	Almendral	Almendral	Badajoz	325	t	5
BGE045633	Rojo de Llerena	Llerena	Badajoz	639	d	5
BGE045668	Raspinegro de Murcia	Águilas	Murcia	6	d	6
BGE045634	Arisnegro velloso	Unknown	Islas Canarias	Unknown	d	7
BGE047518	Blancal de Nules	Nules	Castellón	13	t	8
BGE045673	Alemán grano rojo	Unknown	Islas Baleares	Unknown	d	8
BGE047509	Blanco de Vegadeo	Vegadeo	Asturias	10	t	9

**Table 2:** Metaphase I observations. Number of bound arms per cell, percentage of cells with two univalents (2^I^), and variation in the number of bound arms per cell in plants of 16 Spanish landraces of *T. turgidum* grown during meiosis either in the greenhouse at optimum temperature range (18–22 °C) or in a climatic chamber at 30 °C. Hours of the temperature treatment are also indicated.

Accession	Greenhouse (18–22 °C)			Heat Treatment (30 °C)				Bound Arms/Cell Variation (ii–i)
	Bound Arms/Cell (i)	2^I^ (%)	PMCs	Bound Arms/Cell (ii)	2^I^ (%)	PMCs	Hours at 30 °C	
BGE047504	26.40 + 0.26	1.00	73	23.25 ± 0.16	5.00	105	64	−3.15
BGE047513	27.44 + 0.13	1.00	71	27.32 + 0.17	1.00	100	96	−0.12
BGE045667	27.14 + 0.15	0.00	100	25.05 + 0.36	13.00	100	96	−2.09
BGE045654	27.01 + 0.17	1.00	100	27.08 + 0.17	0.00	100	48	0.07
BGE047500	27.65 + 0.14	0.00	100	27.15 + 0.17	0.00	100	72	−0.5
BGE045665	27.79 + 0.09	0.00	44	27.11 + 0.19	3.00	100	144	−0.68
BGE045640	27.73 + 0.11	0.00	100	26.21 + 0.17	2.00	100	72	−1.52
BGE047514	27.65 + 0.12	0.00	100	26.33 + 0.25	3.00	100	96	−1.32
BGE045675	27.54 + 0.13	0.00	100	26.02 + 0.26	2.00	100	48	−1.52
BGE047501	27.29 + 0.17	0.00	100	26.61 ± 0.10	0.00	100	48	−0.68
BGE045633	27.47 + 0.17	0.00	100	25.95 + 0.27	3.00	100	96	−1.52
BGE045668	26.65 + 0.24	4.00	100	26.72 + 0.21	0.00	100	48	0.07
BGE045634	26.40 + 0.20	1.00	100	26.03 + 0.24	0.00	100	72	−0.37
BGE047518	27.59 + 0.14	0.00	100	24.79 + 0.42	6.00	100	120	−2.8
BGE045673	26.96 + 0.18	2.00	100	25.66 + 0.35	0.00	100	72	−1.3
BGE047509	27.73 + 0.11	0.00	100	27.02 + 0.23	2.00	100	72	−0.71

**Table 3:** The number of bound arms per cell in plants of Spanish landraces of *T. turgidum* subjected or not at heat temperature during premeiosis and meiosis are compared using Mann-Whitney-Wilcoxon tests.

Accession	Heat Treatment	Bound Arms/Cell										W	*p*
		28	27	26	25	24	23	22	21	20	<20		
BGE047504	No	12	26	18	13	4	0	0	0	0	0	655	<0.001
	Yes	2	3	7	14	24	21	13	11	7	3		
BGE047513	No	39	24	8	0	0	0	0	0	0	0	3749	0.49
	Yes	50	37	9	3	1	0	0	0	0	0		
BGE045667	No	37	41	21	1	0	0	0	0	0	0	8644.5	<0.001
	Yes	4	18	22	23	17	8	3	2	3	0		
BGE045654	No	32	43	20	4	1	0	0	0	0	0	4794.5	0.57
	Yes	36	41	18	5	0	0	0	0	0	0		
BGE047500	No	75	17	6	2	0	0	0	0	0	0	6799	<0.001
	Yes	39	41	17	2	1	0	0	0	0	0		
BGE045665	No	36	7	1	0	0	0	0	0	0	0	3177	<0.001
	Yes	40	39	15	4	2	0	0	0	0	0		
BGE045640	No	77	20	2	1	0	0	0	0	0	0	9274	<0.001
	Yes	2	40	36	21	1	0	0	0	0	0		
BGE047514	No	72	22	5	1	0	0	0	0	0	0	8183	<0.001
	Yes	19	29	29	13	9	1	0	0	0	0		
BGE045675	No	63	29	7	1	0	0	0	0	0	0	904	<0.001
	Yes	14	23	30	20	10	3	0	0	0	0		
BGE047501	No	49	35	12	4	0	0	0	0	0	0	6771.5	<0.001
	Yes	23	36	27	8	6	0	0	0	0	0		
BGE045633	No	63	26	9	0	1	1	0	0	0	0	8319.5	<0.001
	Yes	13	22	32	19	10	2	2	0	0	0		
BGE045668	No	28	32	23	13	3	0	1	0	0	0	4896	0.79
	Yes	25	39	24	7	5	0	0	0	0	0		
BGE045634	No	20	33	23	16	5	3	0	0	0	0	5860	0.03
	Yes	9	29	31	22	6	2	1	0	0	0		
BGE047518	No	67	27	5	1	0	0	0	0	0	0	9355.5	<0.001
	Yes	4	15	21	25	15	8	5	3	3	1		
BGE045673	No	31	42	20	6	1	0	0	0	0	0	7212	<0.001
	Yes	14	25	20	15	10	12	4	0	0	0		
BGE047509	No	76	22	1	1	0	0	0	0	0	0	6558.5	<0.001
	Yes	45	28	15	8	4	0	0	0	0	0		

## Data Availability

Not applicable.

## References

[1] Shewry P.R., Hey S.J. (2015). The contribution of wheat to human diet and health. Food Energy Secur..

[2] Beres B.L., Rahmani E., Clarke J.M., Grassini P., Pozniak C.J., Geddes C.M., Porker K.D., May W.E., Ransom J.K. (2020). A Systematic review of durum wheat: Enhancing production systems by exploring genotype, environment, and management (G × E × M) synergies. Front. Plant Sci..

[3] Huang S., Sirikhachornkit A., Su X., Faris J., Gill B., Haselkorn R., Gornicki P. (2002). Genes encoding plastid acetyl-CoA carboxylase and 3-phosphoglycerate kinase of the *Triticum/Aegilops* complex and the evolutionary history of polyploid wheat. Proc. Natl. Acad. Sci. USA.

[4] Dvorak J., di Terlizzi P., Zhang H.B., Resta P. (1993). The evolution of polyploid wheats: Identification of the A genome donor species. Genome.

[5] Sarkar P., Stebbins G.L. (1956). Morphological evidence concerning the origin of the B genome in wheat. Am. J. Bot..

[6] Dvorak J., Zhang H.B. (1990). Variation in repeated nucleotide sequences sheds light on the phylogeny of the wheat B and G genomes. Proc. Natl. Acad. Sci. USA.

[7] Kihara H. (1944). Discovery of the DD-analyser, one of the ancestors of *Triticum vulgare*. Agric. Hortic..

[8] McFadden E.S., Sears E.R. (1946). The origin of *Triticum spelta* and its free-threshing hexaploid relatives. J. Hered..

[9] Naranjo T., Benavente A., Molnár-Láng M., Ceoloni C., Doležel J. (2015). The mode and regulation of chromosome pairing in wheat-alien hybrids (*Ph.* genes, an updated view). Allien Introgression in Wheat. Cytogenetics, Molecular Biology, and Genomics.

[10] Okamoto M. (1957). Asynaptic effect of chromosome V. Wheat Inf. Serv..

[11] Sears E.R., Okamoto M. Intergenomic chromosome relationship in hexaploid wheat. Proceedings of the 10th International Congress of Genetics.

[12] Riley R., Chapman V. (1958). Genetic control of the cytologically diploid behaviour of hexaploid wheat. Nature.

[13] Riley R., Chapman V. (1964). The effect of the deficiency of the long arm of chromosome 5B on meiotic pairing in *Triticum aestivum*. Wheat Inf. Serv..

[14] Riley R., Kempana C. (1963). The homoeologous nature of the non-homologous meiotic pairing in *Triticum aestivum* deficient for chromosome V (5B). Heredity.

[15] Rey M., Martín A.C., Higgins J., Swarbreck D., Uauy C., Shaw P., Moore G. (2017). Exploiting the ZIP4 homologue within the wheat *Ph1* locus has identified two lines exhibiting homoeologous crossover in wheat-wild relative hybrids. Mol. Breed..

[16] Rey M.D., Martin A.C., Smedley M., Hayta S., Harwood W., Shaw P., Moore G. (2018). Magnesium increases homoeologous crossover frequency during meiosis in ZIP4 (*Ph1* gene) mutant wheat-wild relative hybrids. Front. Plant Sci..

[17] Martín A.C., Alabdullah A.K., Moore G. (2021). A Separation-of-function ZIP4 wheat mutant allows crossover between related chromosomes and is meiotically stable. Sci. Rep..

[18] Bennett M.D. (1977). The time and duration of meiosis. Phil. Trans. R. Soc. Lond. B.

[19] Armstrong S.J., Franklin F.C.H., Jones G.H. (2003). A meiotic time-course for *Arabidopsis thaliana*. Sex. Plant Reprod..

[20] Gray S., Cohen P.E. (2016). Control of meiotic crossovers: From double-strand break formation to designation. Annu. Rev. Genet..

[21] De Storme N., Geelen D. (2014). The impact of environmental stress on male reproductive development in plants: Biological processes and molecular mechanisms. Plant Cell Environ..

[22] Fuchs L.K., Jenkins G., Phillips D.W. (2018). Anthropogenic impacts on meiosis in plants. Front. Plant Sci..

[23] Elliott C.G. (1955). The effect of temperature on chiasma frequency. Heredity.

[24] Wilson J.Y. (1959). Temperature effect on chiasma frequency in the bluebell *Endymion nonscriptus*. Chromosoma.

[25] Lin Y.J. (1982). Temperature and chiasma formation in *Rhoeo spathacea* var. variegata. Genetica.

[26] Dowrick G.J. (1957). The influence of temperature on meiosis. Heredity.

[27] Si W., Yuan Y., Huang J., Zhang X., Zhang Y., Zhang Y., Zhang Y., Tian D., Wang C., Yang Y. (2015). Widely distributed hot and cold spots in meiotic recombination as shown by the sequencing of rice F2 plants. New Phytol..

[28] Phillips D., Jenkins G., Macaulay M., Nibau C., Wnetrzak J., Fallding D., Colas I., Oakey H., Waugh R., Ramsay L. (2015). The effect of temperature on the male and female recombination landscape of barley. New Phytol..

[29] Lloyd A., Morgan C., Franklin C., Bomblies K. (2018). Plasticity of meiotic recombination rates in response to temperature in *Arabidopsis*. Genetics.

[30] Porter J.R., Gawith M. (1999). Temperatures and the growth and development of wheat: A review. Eur. J. Agron..

[31] Fischer R.A., Maurer R. (1976). Crop temperature modification and yield potential in a dwarf spring wheat. Crop. Sci..

[32] Wardlaw I.F., Dawson I.A., Munibi P., Fewster R. (1989). The tolerance of wheat to high temperatures during reproductive growth. I. Survey procedures and general response patterns. Aust. J. Agric. Res..

[33] Saini H.S., Aspinall D. (1982). Abnormal sporogenesis in wheat (*Triticum aestivum* L.) induced by short periods of high temperature. Ann. Bot..

[34] Draeger T., Moore G. (2017). Short periods of high temperature during meiosis prevent normal meiotic progression and reduce grain number in hexaploid wheat (*Triticum aestivum* L.). Theor. Appl. Genet..

[35] Bennett M.D., Chapman V., Riley R. (1971). The duration of meiosis in pollen mother cells of wheat, rye and Triticale. Proc. R. Soc. Lond. B.

[36] Bennett M.D., Smith J.B., Kemble R. (1972). The effect of temperature on meiosis and pollen development in wheat and rye. Can. J. Genet. Cytol..

[37] Bennett M.D., Rao M.K., Smith J.B., Bayliss M.W. (1973). Cell development in the anther, the ovule, and the young seed of *Triticum aestivum* L. var. Chinese Spring. Phil. Trans. R. Soc. B.

[38] Bayliss M.W., Riley R. (1972). An analysis of temperature-dependent asynapsis in *Triticum aestivum*. Genet. Res..

[39] Rezaei M., Arzani A., Sayed-Tabatabaei B.E. (2010). Meiotic behaviour of tetraploid wheats (*Triticum turgidum* L.) and their synthetic hexaploid wheat derivates influenced by meiotic restitution and heat stress. J. Genet..

[40] Bayliss M.W., Riley R. (1972). Evidence of premeiotic control of chromosome pairing in *Triticum aestivum*. Genet. Res..

[41] Morais L., Queiroz A., Viegas W., Roca A., Naranjo T. (1992). Synaptonemal complex formation and metaphase I bond distribution at low temperatures in nullisomic 5D-Tetrasomic 5B hexaploid wheat. Genome.

[42] Draeger T., Martin A.C., Alabdullah A.K., Pendle A., Rey M.D., Shaw P., Moore G. (2020). *Dmc1* is a candidate for temperature tolerance during wheat meiosis. Theor. Appl. Genet..

[43] Ruiz M., Giraldo P., Royo C., Carrillo J.M. (2013). Creation and validation of the Spanish durum wheat core collection. Crop. Sci..

[44] Ruiz M., Giraldo P., Royo C., Villegas D., Aranzana M.J., Carrillo J.M. (2012). Diversity and genetic structure of a collection of Spanish durum wheat landraces. Crop. Sci..

[45] Sybenga J. (1992). Cytogenetics in Plant Breeding.

[46] Saini H.S., Sedgley M., Aspinall D. (1984). Developmental anatomy in wheat of male-sterility induced by heat-stress, water deficit or abscisic-acid. Aust. J. Plant Physiol..

[47] Barnabás B., Jäger K., Fehér A. (2008). The effect of drought and heat stress on reproductive processes in cereals. Plant Cell Environ..

[48] Vierling E. (1991). The roles of heat shock proteins in plants. Ann. Rev. Plant Physiol. Plant Mol. Biol..

[49] Holmberg N., Bulow I. (1998). Improving stress tolerance in plants by gene transfer. Trends Plant. Sci..

[50] Mullarkey M., Jones P. (2000). Isolation and analysis of thermotolerant mutants of wheat. J. Exp. Bot..

[51] Chauhan H., Khurana N., Agarwal P., Khurana J.P., Khurana P. (2013). A seed preferential heat shock transcription factor from wheat provides abiotic stress tolerance and yield enhancement in transgenic *Arabidopsis* under heat stress environment. PLoS ONE.

[52] Lu P.P., Zheng W.J., Wang C.T., Shi W.Y., Fu J.D., Chen M., Chen J., Zhou Y.B., Xi Y.J., Su Z.S. (2018). Wheat Bax Inhibitor-1 interacts with TaFKBP62 and mediates response to heat stress. BMC Plant Biol..

[53] Telfer P., Edwards J., Norman A., Bennett D., Smith A., Able J.A., Kuchel H. (2021). Genetic analysis of wheat (*Triticum aestivum*) adaptation to heat stress. Theor. Appl. Genet..

[54] RStudio Team 2020 RStudio: Integrated Development for R. RStudio, PBC, Boston, MA. http://www.rstudio.com/.

